# Implicit Stochastic Gradient Descent Method for Cross-Domain Recommendation System

**DOI:** 10.3390/s20092510

**Published:** 2020-04-29

**Authors:** Nam D. Vo, Minsung Hong, Jason J. Jung

**Affiliations:** 1 Department of Computer Engineering, Chung-Ang University, 84 Heukseok, Seoul 156-756, Korea; 2 Big Data Research Group, Western Norway Research Institute, Box 163, NO-6851 Sogndal, Norway

**Keywords:** cross-domain, user rating consolidation, recommendation system, inner approximation, implicit update, convex optimization

## Abstract

The previous recommendation system applied the matrix factorization collaborative filtering (MFCF) technique to only single domains. Due to data sparsity, this approach has a limitation in overcoming the cold-start problem. Thus, in this study, we focus on discovering latent features from domains to understand the relationships between domains (called domain coherence). This approach uses potential knowledge of the source domain to improve the quality of the target domain recommendation. In this paper, we consider applying MFCF to multiple domains. Mainly, by adopting the implicit stochastic gradient descent algorithm to optimize the objective function for prediction, multiple matrices from different domains are consolidated inside the cross-domain recommendation system (CDRS). Additionally, we design a conceptual framework for CDRS, which applies to different industrial scenarios for recommenders across domains. Moreover, an experiment is devised to validate the proposed method. By using a real-world dataset gathered from Amazon Food and MovieLens, experimental results show that the proposed method improves 15.2% and 19.7% in terms of computation time and MSE over other methods on a utility matrix. Notably, a much lower convergence value of the loss function has been obtained from the experiment. Furthermore, a critical analysis of the obtained results shows that there is a dynamic balance between prediction accuracy and computational complexity.

## 1. Introduction

Recent achievements in the Internet and computing technologies have made it possible for organizations to collect, store, and process large amounts of data. These data contain detailed information related to the behaviors of users. Accurately, they represent the set of user evaluations for specific items. For example, Amazon (https://www.amazon.com/) collects information about the user’s habits shopping-wise, or even regarding surfing on their website. Netflix (https://www.netflix.com/) also has substantial data related to the subject of movies. These data are beneficial for recommending useful decisions when supporting their clients. In this scenario, each firm designs a unique and maximally efficient system that can recommend as pleasant as possible items to its customers [1]. Nevertheless, not all users give ratings for items that they like or dislike. This limitation causes the fragmentation of the dataset obtained from the user, which is called data sparsity. In the real-life, a dataset is sparse at around 0.05% [2]. Therefore, the system can not produce useful recommendations when a new user or item has entered the system due to the insufficient previous ratings. This problem is named the cold-start [3], which is the most challenging issue for researchers to overcome. The cold-start problem is that problem wherein a system is not able to recommend items to users. Every recommender system is required to build a user’s profile by considering his/her preferences and likes. The user’s profile is developed by considering his/her activities and behaviors being perform with the system. Based on user’s previous history and activities, the system makes decisions and recommends items consequently. Many investigations have been proposed to solve the cold-start problem by locating extra information among the intradomain objects to imply the association between a user and item [4,5,6,7]. Nevertheless, we cannot always obtain this kind of extra useful information.

On the other hand, the cold-start problem in insufficient data in one domain can be solved if another domain has relatively abundant data [8]. In other words, since there exists either implicitly or explicitly correlated between domains, we could overcome the cold-start problem by grouping multiple domains. In particular, the latent features existing among domains may improve recommendation accuracy. By this approach, a recommendation system can be built to exploit valuable information from one domain to contribute to another domain. These systems are known as cross-domain recommendation systems (CDRSs) [9]. In CDRSs, one of the most popular and efficient methods that has been used is matrix factorization collaborative filtering (MFCF) [10]. This method could handle both two major problems regarding two directions of CDRS development. The first direction focuses on collecting preference data from users and items from all domains. Oppositely, the CDRS in the second direction aims to connect domains based on other information, such as the properties of items or the social relations of users [11]. The preference data are exclusively focused on our research, since they is not affected by other information yet can be applied widely. Zhang et al. (2018) classified the preference-based CDRS into two groups: the first one is the situation in which there are no common areas between domains, while the other group has at least a partial overlap between domains [12]. In this paper, we concentrate on the first class of CDRS, where there is not any overlap among domains, since this situation is prevalent in real life. Regarding this type of CDRS, the existing method has used shared information from the items and users in domains [13]. Notably, similar information related to the item’s contents and user’s preferences was extracted from all domains to build the group-level knowledge, which is used for the utility matrices afterward [14]. Nevertheless, there are some limitations to this approach, since it is unsteady to transfer knowledge from one domain to the other. This unstable state will adversely affect the performance of the recommendation system [15].

Differently from previous works, to overcome the limitations mentioned above, we propose an efficient framework for a cross-domain recommendation system. In this framework, multiple domains that are presented by matrices are consolidated into one. Then we apply the MFCF to predict the unknown ratings from user to item. In this way, it is possible to extract the latent features from the user-group and item-group. An implicit update technique is adopted while optimizing the objective function to increase prediction accuracy. Additionally, the optimization convergence is significantly improved.

The main contributions of this study are as follows:

- We propose an efficient framework for a cross-domain recommendation system based on a constrained optimization model. In our model, the optimal solution and computation time are simultaneously taken into consideration.
- We devise an approximation algorithm that is suitable for objective function optimization in a cross-domain related problem. In particular, an implicit updating technique is applied to improve convergence time.
- We conduct extensive experiments on two real-world datasets to validate the effectiveness and efficiency of our method. The results demonstrate that the proposed framework can achieve better performance in comparison with the previous approach.

The remainder of the paper is organized as follows. Section 2 explains the background knowledge of MFCF in a single domain and reviews literature related to CRDS. Section 3 formally defines the problem formulation. In Section 4, we present our conceptual framework for CDRS. Section 5 presents an experiment. Finally, we draw conclusions and suggest directions for future study in Section 6.

## 2. Related Work and Background

### 2.1. Related Work

Recent researchers have studied cross-domain related work, as mentioned in [11,16,17], wherein there are two types of cross-domain recommended tasks. The first task is to use the information of the source domain to enhance the quality of the target domain recommendation [18,19,20]. Karatzoglou et al. used a machine learning method to transfer dense knowledge from the source domain to the target areas, which is much more sparse [21]. Enrich et al. used the user tags as connections between multiple domains, from which they learn the users’ rating models to gain performance in the target domain [22]. The second task is recommending items in separate domains concurrently. They proposed a method for creating a rating matrix, which is the multidisciplinary shared latent factor [23,24]. Shi et al. [25] used the user-generated tags to calculate the similarity between cross-domain users and items, respectively, and then integrated these similarities into a matrix factorization model to improve the recommended accuracy. Gao et al. presented the clustering latent factor model based on a joint non-negative matrix framework [26].

For recommendation using matrix factorization, work was done by Gogna et al. [27]. They proposed a matrix completion framework that can be implemented in different domains. Zhenzhen et al. presented a cross-domain recommendation algorithm to overcome cold-start and sparsity problems and mentioned that this could be extended to consider temporal dynamics, as user preferences may change over time [28]. A cross-domain collaborative framework for recommending the venue proposed by Farseev et al. [29] is not able to address the cold-start problem. Loni et al. [30] presented a cross-domain factorization machine that can exploit additional knowledge from an auxiliary domain by encoding specific knowledge from a domain in terms of the real-valued feature vector.

In this study, we apply the MFCF for multiple domains using an updated technique to increase the convergence time of the objective function. Additionally, the implicit stochastic gradient descent-based algorithm is utilized to apply to the cross-domain recommendation system.

### 2.2. Background

In a single domain, let us suppose there are M users and N items. The relationship between the users and the items is presented by the user-item rating matrix $Y\in R^{M\times N}$, called utilitymatrix. Any rating $r_{ij}$ in Y is subject to $r_{ij}\in \left\{1,2,3,4,5,?\right\}$, where “?” represents missing value. To predict the missing values, users and items are clustered. The utility matrix Y can be factorized into two matrices $Y\approx \hat{Y}=XW^{T}$, where $X\in R^{M\times K}$ is the user-group membership matrix, and $W\in R^{N\times K}$ is the item-group membership matrix.

**Figure 1:**
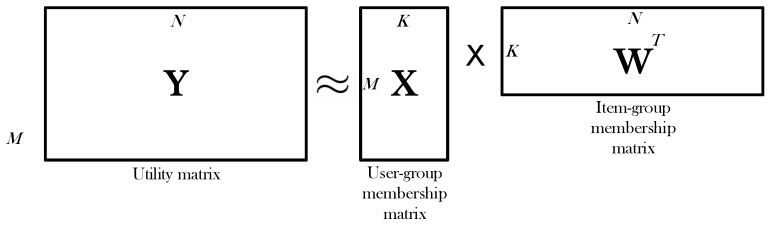
Decomposing utility matrix into two matrices.

Figure 1 represents the matrix factorization, in which the full utility matrix Y is decomposed into two matrices X and W, where *K* is much smaller than *M*, *N*. Each row in X represents a *user profile*x, and each column in W denotes an *item profile*w. On the other hand, the *i*-th item and the *j*-th user are represented by the *i*-th and *j*-th rows of the two matrices as $W_{i*}$ and $X_{j*}$. After matrix factorization, the users and items are mapped to a latent factor feature of a lower dimensionality *K*.

To predict the missing values in the utility matrix, the low-rank matrix factorization is approximated as an optimization problem given by

(1) $$\min_{X,W}\;L\left(f\left(X,W\right),Y\right)+\lambda R\left(X,W\right),$$

where L is the loss function of the predicted ratings f(X,W) and the original ratings Y, R(X,W) is the regularization term, and λ is the regularization tradeoff parameter. λR(X,W) is regularization component to avoid overfitting. Regarding probabilistic matrix factorization (BMF) [31,32],  the objective function to measure the loss with regularization terms and a Frobenius norm is expressed as

(2) $$J\left(X,W\right)=\frac{1}{2}∥I⊙\left(Y-XW^{T}\right){∥}_{F}+\frac{\lambda }{2}{∥X∥}_{F}+\frac{\lambda }{2}{∥W∥}_{F},$$

where I is the rating indicator matrix, $I_{ij}\in \left\{0,1\right\}$. $I_{ij}=1$ indicates that the rating is observed, or $I_{ij}=0$ otherwise. ⊙ denotes the Hadamard product [33] of the matrices.

## 3. Problem Formulation

### 3.1. Definition of User-Preference Matrix

Let $D_{l}$ with l∈(1,L) be the user-preference matrix with response to *l*-th domain. Then, the entries of $D_{l}$ which are denoted by ${\left(D_{l}\right)}_{ij}$ indicate the ratings of the *i*-th user for the *j*-th items of set $D_{l}$.

By U we denote the set of all users that exist in multiple domains:

(3) $$U=\{U^{D_{1}},U^{D_{2}},\cdots ,U^{D_{L}}\},$$

where $U^{D_{l}}$ is the sets of users in *l*-th domain. Although these matrices are overlapping or nonoverlapping, the matrix V, which is built from the consolidation of matrices $D_{1},$$D_{2}$, ...$D_{L}$ has the number of rows given by |U|. Given a user $U_{u},\;u\in \left\{1,2,\cdots ,|U|\right\}$ in U, the matrix $D_{l}$ can be rewritten as follows:

(4) $$D_{l}={\left[{\left(d_{1}^{D_{l}}\right)}^{T},\cdots ,{\left(d_{u}^{D_{l}}\right)}^{T},\cdots ,{\left(d_{\left|U\right|}^{D_{l}}\right)}^{T}\right]}^{T},$$

where the row vector $d_{u}^{X},\;X\in \left\{D_{1},D_{2},D_{L}\right\}$, contains the corresponding rating values of all items in $X\in \{D_{1},D_{2},D_{L}\}$, voted by user $U_{u}$. Clearly, $d_{u}^{X}=0$, if $U_{u}\in U\backslash U^{X}$.

For generality, all the user’s ratings can be described by the following matrix V:

(5) $$V=[D_{1}\;D_{2}\cdots D_{L}].$$

Matrix V is the expandable matrix since its dimensionality increases when adding new items and users to the data. We denote the transpose matrix of V by $V^{T}$.

A column vector b is given as

(6) $$\begin{array}{cc} b & =\left[\begin{array}{c} b_{1}\\ \;b_{2}\\ \;\vdots \\ \;b_{n} \end{array}\right], \end{array}$$

where *n* denotes the number of rows (the number of users) of V. Each entry $b_{i}$ is the inverse of a square root of the element $a_{ii}$ in the $VV^{T}$ diagonal. Therefore, $b_{i}$ is as follows:

(7) $$b_{i}=\frac{1}{\sqrt{a_{ii}}}.$$

Then it is possible to write formula (6) in the following form

(8) $$\begin{array}{cc} b & =\left[\begin{array}{c} \frac{1}{\sqrt{a_{11}}}\\ \;\frac{1}{\sqrt{a_{22}}}\\ \;\vdots \\ \;\frac{1}{\sqrt{a_{nn}}} \end{array}\right]. \end{array}$$

By $b^{T}$ we denote the transform matrix of b, and matrix $B=bb^{T}$. In this regard, a similarity matrix S can be written as follows:

(9) $$S=(VV^{T})⊙B.$$

This operator is a Hadamard product, in which each element p, q in S is the product of elements p and q of the original two matrices $\left(VV^{T}\right)$ and B. After this operator, S will be the symmetric matrix with rows and columns being users, in which each element $S_{ij}$ is the cosine of the angle between two vectors $u_{i}$ and $u_{j}$, where $u_{i}$ and $u_{j}$ are the *i*-th and *j*-th rows of V, respectively.

**Remark** **1.** *By considering the preference vectors $u_{i}$ and $u_{j}$, i≠j, the similarity between i-th and j-th users is properly given by $S_{ij}$; i.e.,*

(10) $$S_{ij}=cos\left(u_{i},u_{j}\right)=\frac{u_{i}u_{j}^{T}}{\left|\right|u_{i}{\left|\right|}_{2}\cdot ||u_{j}{\left|\right|}_{2}}.$$

Given by the *i*-th row and *j*-th column entry of matrix, $VV^{T}$ is equal to $u_{i}u_{j}^{T}$, with the corresponding entry $B_{ij}$ of matrix B equivalent to

(11) $$B_{ij}=\frac{1}{\left|\right|u_{i}{\left|\right|}_{2}\cdot ||u_{j}{\left|\right|}_{2}}.$$

Therefore, (9) is considered as a generalized formulation to derive the similarity matrix among users with respect to all items.

Similarly, we can find the item-similarity matrix as follows.

(12) $$K=(V^{T}V)⊙C,$$

where $C=cc^{T}$, c is a row vector c = $\left[c_{1},c_{2}\cdots ,c_{m}\right]$ with $c_{m}$ denoting the inverse of a square root of the element $d_{mm}$ in the $V^{T}V$ diagonal of V. Therefore, $c_{m}$ is as follows:

(13) $$c_{m}=\frac{1}{\sqrt{d_{mm}}}.$$

Now we will factorize matrix V. As mentioned in the previous Section, the user *n* gives a rating to the item *m* that can be approximated as $y_{mn}=x_{m}^{T}w_{n}$. However, the actual ratings have biases for users or/and items, since users tend to rate the items according to their rating behaviors, resulting in ratings that may be larger or smaller than the actual values the items receive. We use bias to overcome this problem. By $\mu_{m}$ and $\mu_{n}$, we denote biases for item m and user n, respectively. Then the rating is approximated by

(14) $$y_{mn}\approx x_{m}w_{n}+\mu_{m}+\mu_{n}+\mu ,$$

where μ is median value of all ratings.

Therefore, the loss function (2) can be written as

(15) $$\begin{array}{c} L\left(X,W,\mu_{m},\mu_{n}\right)=\frac{1}{2s}\sum_{n=1}^{N}\sum_{m=1}^{M}{\left(x_{m}w_{n}+\mu_{m}+\mu_{n}+\mu -y_{mn}\right)}^{2}+\\ +\frac{\lambda }{2}{\left(||X|\right|}_{F}^{2}+{\left||W|\right|}_{F}^{2}+\left|\right|\mu_{m}{\left|\right|}_{F}^{2}+\left|\right|\mu_{n}{\left|\right|}_{F}^{2}). \end{array}$$

In the previous works, this loss function is solved by optimizing one of the pairs ($X,\mu_{m}$) and ($W,\mu_{n}$) respectively, while fixing the other pair. This process is repeated until the loss function converges. This push–pull [34] gradient method will get a sub-optimal solution [35]. In contrast with the earlier investigations, we will solve this loss function by optimizing ($X,W,\mu_{m},\mu_{n}$) simultaneously.

### 3.2. Algorithm for Prediction Error Minimization

In this section, we investigate the following joint design problem for prediction error model minimization:

(16) $$\;\underset{X,W,\mu_{m},\mu_{n},t}{\mathrm{minimize}}\;L=\frac{1}{2s}\sum_{n=1}^{N}\sum_{m=1}^{M}t_{mn}^{2}+\frac{\lambda }{2}{\left(||X|\right|}_{F}^{2}+{\left||W|\right|}_{F}^{2}+\left|\right|\mu_{m}{\left|\right|}_{F}^{2}+\left|\right|\mu_{n}{\left|\right|}_{F}^{2}),$$

where $t≜\left[t_{mn}\right],\;\forall m\in \left\{1,\cdots ,M\right\},\;\forall n\in \left\{1,\cdots ,N\right\}$, with $t_{mn}$ satisfying the following constraint:

(17) $$x_{m}w_{n}+\mu_{m}+\mu_{n}+\mu -y_{mn}\leq t_{mn}.$$

Although the objective function in (16) is a quadratic representative, which is convex, constraint (17) is still non-convex. To efficiently solve this problem, we derive a successive convex program based on an inner approximation method [36] as follows:

It is observed that (17) is equivalent to the convex constraint:

(18) $$\sum_{k=1}^{K}u_{mnk}^{2}\leq t_{mn}-\mu_{m}-\mu_{n}-\mu +y_{mn}$$

with the following constraint imposed

(19) $$x_{mk}w_{nk}\leq u_{mnk}^{2}.$$

However, constraint (19) is still non-convex. Inspired from ([37], Lemma 1), (19) can be approximated as

(20) $$\frac{{\bar{w}}_{nk}}{2{\bar{x}}_{mk}}x_{mk}^{2}+\frac{{\bar{x}}_{mk}}{2{\bar{w}}_{nk}}w_{nk}^{2}\leq u_{mnk}^{2},$$

which is convex as a second order cone constraint. Here, ${\bar{x}}_{mk}$ and ${\bar{w}}_{nk}$ are respectively the values of $x_{mk}$ and $w_{nk}$ at the previous iteration. Therefore, the successive convex program is formulated as

(21a) $$\begin{array}{cccc} \; & \;\underset{X,W,\mu_{m},\mu_{n},t,u}{\mathrm{minimize}} & \; & L=\frac{1}{2s}\sum_{n=1}^{N}\sum_{m=1}^{M}t_{mn}^{2}+\frac{\lambda }{2}{\left(||X|\right|}_{F}^{2}+{\left||W|\right|}_{F}^{2}+\left|\right|\mu_{m}{\left|\right|}_{F}^{2}+\left|\right|\mu_{n}{\left|\right|}_{F}^{2}) \end{array}$$

(21b) $$\begin{array}{cccc} \; & \mathrm{subject}\;\mathrm{to} & \; & (18),(20). \end{array}$$

It is realized that the problems in (21) can be efficiently solved per iteration by the existing solver (e.g., SPDT3 [38], MOSEK [39], or SeDuMi [40]), so that we obtain at least a locally optimal solution at the convergence. The algorithm for solving problem in (21) is briefly described in Algorithm 1.
**Algorithm 1:** Iterative algorithm for the prediction error optimization.1 **Initialization:** Set $L^{min}$ := +*∞*, $\left(x^{*},t^{*},w^{*},u^{*}\right)$ := 02 **for** each *k*
∈K
do {solving subproblem (19)}3  **Generating an initial points:** Set *k*:= 0 and solve (20) to generate ($x^{\left(0\right)},\mu_{m}^{\left(0\right)},w^{\left(0\right)},\mu_{n}^{\left(0\right)}$).4  **repeat**5   Solve (21) to obtain ($x^{*},\mu_{m}^{*},w^{*},\mu_{n}^{*}$) and $L^{\left(k+1\right)}$.6   Update ($x^{\left(k+1\right)},\mu_{m}^{\left(k+1\right)},w^{\left(k+1\right)},\mu_{n}^{\left(k+1\right)}$):=($x^{*},\mu_{m}^{*},w^{*},\mu_{n}^{*}$).7   Set k=k+1.8  **until** Convergence9  **if**
$L^{\left(k\right)}<L^{min}$
then10   Update $L^{min}$ := $L^{\left(k\right)}$ and ($x^{*},\mu_{m}^{*},w^{*},\mu_{n}^{*}$):=($x^{\left(k\right)},\mu_{m}^{\left(k\right)},w^{\left(k\right)},\mu_{n}^{\left(k\right)}$).11  **end if**12 **end for**

In Algorithm 1, we use the implicit update technique to increase the convergence speed. The initial values of x and w are random. For the practical implementation, Algorithm 1 terminates upon reaching $L^{\left(k+1\right)}-L^{\left(k\right)}<\varepsilon$ after a finite number of iterations [41].

## 4. CDRS Framework

In this section, we propose a conceptual framework for a cross-domain recommender system that applies the proposed method [42]. When businesses launch multiple products or services, a mass number of data are processed to make the recommendations to clients. These data are heterogeneous and imbalanced, since their sources are from different domains. Data from users, such as ratings, number of likes, and website surfing history, are collected, clustered, and stored into the database. The cross-domain recommendation system engine will process these data to build the model. A set of parameters could be adjusted at this stage to obtain the best accuracy. The system output is the user-preferences prediction that is used to recommend items to the customers.

**Figure 2:**
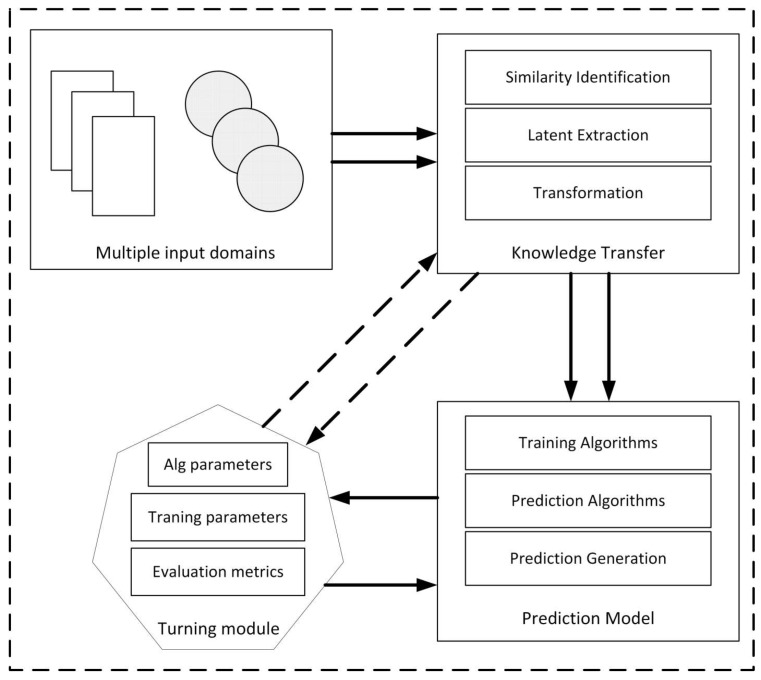
Conceptual framework for cross-domain recommendation system.

Particularly, according to the Figure 2, multiple datasets from various domains are preprocessed in a knowledge transfer module. Here, these data will have similarities identified and latent features extracted, and we will perform knowledge transformation. Then in the next phase, the prediction model will analyze all the information exported from the preprocessing phase in order to apply the appropriate algorithms for training and prediction generation. In this phase, most parameters are turned repeatedly to choose the best set for maximizing the whole system’s accuracy. By this workflow, a CDRS can deal with heterogeneous input data and produce recommendation items in various scenarios.

## 5. Experiments

In this section, we report experiments done to evaluate the recommendation quality of the proposed recommendation model against some baseline state-of-the-art recommendation techniques.

### 5.1. Dataset

To better illustrate our method, this section outlines a small-scale example. There were two datasets used: Movielens (https://movielens.org/) and Amazon Food (https://www.kaggle.com/snap/amazon-fine-food-reviews). The statistical information for these datasets is presented in Table 1.

As shown in Table 1, the movielens100k dataset includes 943 users with 90,570 ratings for 1675 items. Therefore, its sparsity is extremely high (0.057%). Similarly, the Amazon food dataset is sparse, at around 0.058%. This sparsity is natural with respect to real-world situations in recommendation services [43]. The remaining unknown ratings are a big challenge for the recommender system to predict.

We chose three other related algorithms to compare with the proposed algorithm:

- The rating matrix generative model (RMGT): [23] one of the most popular algorithms for testing cross-domain recommended performance.
- The singular value decomposition-based MF (SVD) [44].
- The SVD++-based MF (SVD++) [45] is an extension of the SVD considering implicit ratings.

For each algorithm, we used gradient descent and implicit stochastic gradient descent, respectively, for optimization.

### 5.2. Evaluation Metric

We adopt the mean square error (MSE) to measure the accuracy of predicted ratings, which measures the sum of squared distances between our target ratings and predicted values. MSE is defined as follows:

(22) $$\mathrm{MSE}=\frac{\sum_{i=1}^{n}{\left(y_{i}-y_{i}^{p}\right)}^{2}}{n}.$$

Additionally, we use mean absolute error (MAE) which has frequently been used to compare prediction errors of recommendation methods. This measurement is defined as follows:

(23) $$\mathrm{MAE}=\frac{\sum_{i=1}^{n}\left|y_{i}-y_{i}^{p}\right|}{n},$$

where *n* denotes the number of tested ratings, $y_{i}$ is real ratings, and $y_{i}^{p}$ is predicted ratings. This approach is used because the predicted rating values create an ordering across the items in which the predictive accuracy can also be used to measure the ability of a recommendation system to rank items with respect to user preference [46].

We use *k*-fold cross-validation to split the dataset. A *k*-fold cross-validation is where a given dataset is split into a *k* number of sections/folds where each fold is used as a testing set at some point. To select a proper *k* is important since a poorly chosen value may cause a misrepresentation of the methods. In this experiment, *k* is set as 10, because 5 and 10 have empirically shown to yield test error rate estimates that suffer neither from excessively high bias nor very high variance, according to [47]. Here, the dataset is split into ten folds. In the first iteration, the first fold is used to test the model, and the rest is used to train the model. In the second iteration, the second fold is used as the testing set, while the rest serves as the training set. This process is repeated until each fold of the ten folds has been used as the testing set. As we repeat the process *k* times, we get *k* times mean square error (MSE). $MSE_{1}$, $MSE_{2}$, …$MSE_{k}$, so *k*-fold cross-validation error is computed by taking average of the MSE over *k* folds.

### 5.3. Baseline

A matrix factorization method is applied to solve the problem in (15). Eventually, we have to optimize the loss function L. An optimized method based on the gradient descent algorithm is used to solve this problem. Notably, four variables will be separated into two pairs. For each iteration, one of the pairs is kept constant, while the other is optimized [48]. This process repeats sequentially until convergence is achieved based on the push–pull gradient. After convergence, the sub-optimal solution can be obtained. This solution is used as a baseline.

### 5.4. Experiment Parameters

Two optimization methods are used for comparison: gradient descent (GD) and implicit stochastic gradient descent (ISGD) [49,50]. The set of parameters is presented in Table 2. We have chosen these parameters based on a series of empirical tests.

### 5.5. Evaluation and Discussions

Now we solve the problem in this paper by optimizing all the variables simultaneously. The implicit stochastic gradient descent (ISGD) method is applied. Firstly, it is necessary to transform the original problem in (15) into the convex problem [51] formulated in (21). The parameters listed in Table 2 are the same as the baseline case. To deal with a vast quantity of variables, it is required to apply some techniques for accelerating convergence rate. Algorithm 1 shows the updating step in each iteration.

**Figure 3:**
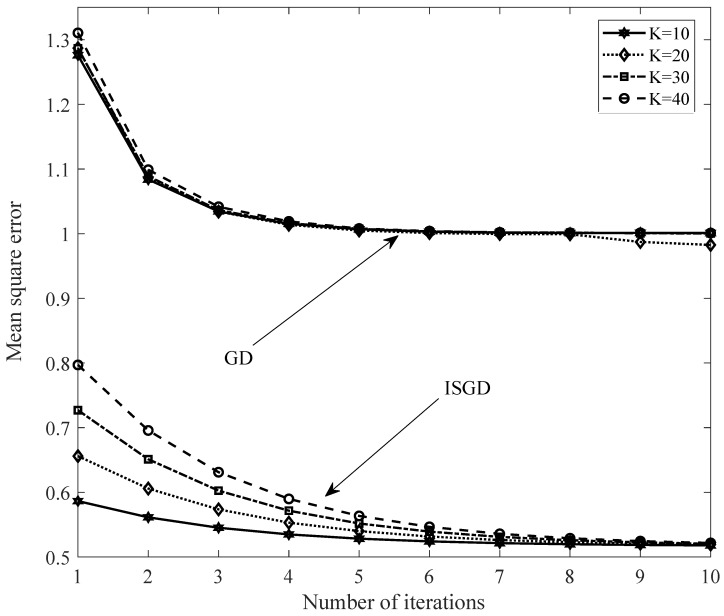
Typical convergence rate of GD and ISGD with varieties of *K*.

Figure 3 shows the typical convergence behavior of the algorithms for the loss function minimization problem. As a result, ISGD needs only a few iterations to reach the convergence value. Moreover, its convergence value is much lower in comparison with the baseline.

When *K* varies from 10 to 40, as shown in Figure 3. The slope of the ISGD convergence line also changes accordingly. When *K* is more extensive, this slope also increases. This leads to the initial value of the objective function also increasing significantly. The results showed the larger the *K* selected, the higher the objective value obtained at the first iteration. When *K* is larger, the dimensions of x,w increase accordingly. This leads to an increase in the number of elements in x,w that makes their values larger. Finally, the value of the objective function will be larger. However, the convergence value is approximately the same.

**Figure 4:**
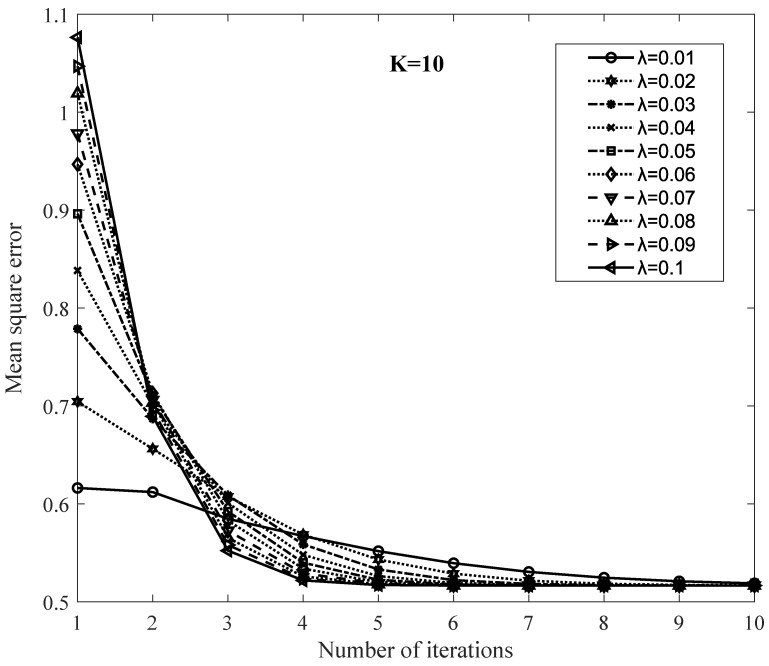
Typical convergence rate of ISGD with varieties of λ.

Let *K* be 10; the results according to changing the value of λ from 0.01 to 0.1 are shown in Figure 4. It shows the difference in the convergence rates when we change the regularization parameter λ. When λ is small, the objective value is obtained as a small value at the first iteration, and the convergence rate is slow. Nevertheless, with the higher λ is selected, a higher objective value is obtained at the first iteration accordingly, and the convergence value is reached faster. When K is increased (e.g., 20, 30, 40) and λ value is set as the highest value (0.1), the initial objective value is much larger since it is affected by two factors, and the convergence value is reached faster.

We recognize that the parameter *K* is used to adjust the approximation process. It acts the role of the dimension for approximation. The bigger *K* is the more accurate approximation. Nevertheless, when *K* increases, the value of the objective function will increase accordingly. It will be a penalty since it has a norm of x and w. This leads to a trade-off problem between the MSE and the computation complexity. *K* can not be so large, and the MSE has to be as small as possible.

**Figure 5:**
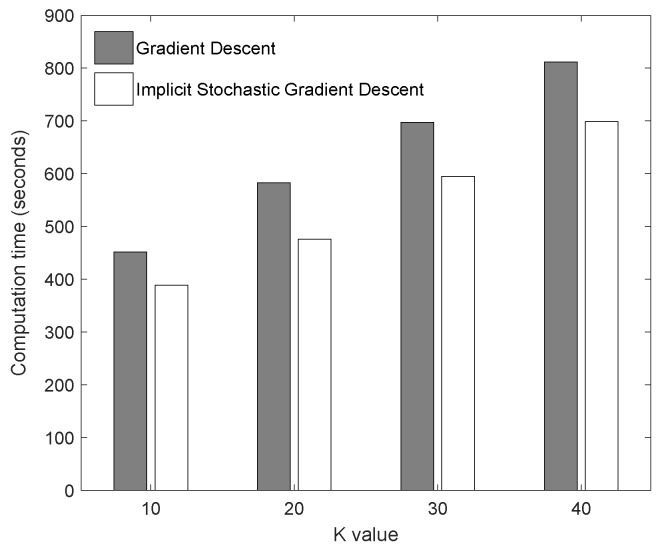
Computation time comparison between two methods.

Regarding convergence time, we have measured the time until convergence between GD and ISDG. The results are shown in Table 3. In Figure 5, we can notice that the proposed method shows efficiency in terms of reducing computation time. On average, computation time has been reduced 15.2%.

**Figure 6:**
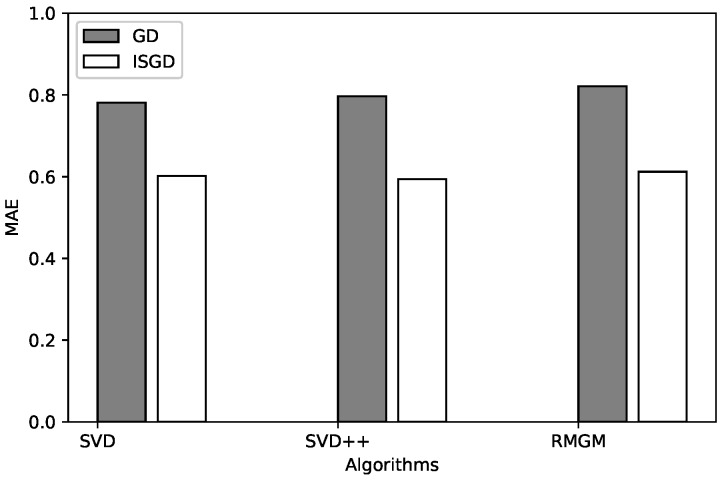
Comparison of MAE with other techniques.

Furthermore, the proposed method shows a significant result regarding prediction accuracy. Table 4 and Figure 6 show an MAE comparison between our method and other techniques. It shows that the effect of the method in this paper is better than that of other comparison methods on all tests. That is, the experimental result shows that using the ISGD technique to optimize the objective function in MFCF improves the performance of the cross-domain recommendation system.

When K varies from 10 to 40, the implicit update techniques show its efficiency to increase the convergence time. Unfortunately, the objective function has a norm of x and w, which can lead to a trade-off problem between the MSE and the computation time. Additionally, our goal is to make the MSE to be as small as possible, so *K* can not be so large. This issue will be the limitation of our paper. We have to make a balance between the accuracy of the recommender system and the computation time.

## 6. Conclusions and Future Works

In this paper, we proposed a new method to consolidate multiple matrices from multiple domains for building a cross-domain recommendation system. After the consolidation, the matrix was factorized by using MFCF. The problem was to maximize the accuracy of the prediction of unknown ratings of users. To address the design problem, we transformed the original problem into sub-problems of lower dimensions. Then the iterative algorithm was proposed based on the inner approximation method to solve the sequence of convex programs. We applied the implicit stochastic gradient descent method for implicit updating each iteration. Our method with realistic parameters monotonically improved the objective function, and the convergence to a stationery point is guaranteed. Through the experiment, we demonstrated the usefulness of our approach in improving the accuracy of the CDRS.

As future work, we plan to consider using multiple data that have different distributions and attributes to test the performance of a cross-domain recommendation system. Based on this way, we can investigate the appropriate set of parameters for each specific type of data or type of domain in general.

## Figures and Tables

**Table 1:** Statistics of datasets.

	Movielens100k	Amazon Food
#user	943	1072
#item	1675	1819
#rating	90,570	113,895
rating range	1–5	1–5

**Table 2:** Experiment parameters.

Parameters	Values
Regularization parameter λ	0.01–0.1
*K*	10–50
Learning rate	50
Initial value of w,x	random
Number of iterations	10

**Table 3:** Computation time comparison (seconds).

K Value	GD Method	ISGD Method
K = 10	452	389
K = 20	583	476
K = 30	697	595
K = 40	812	699

**Table 4:** Comparison of MAE with other techniques.

	SVD_GD	SVD_ISGD	SVD++_GD	SVD++_ISGD	RMGM_GD	RMGM_ISGD
MAE	0.7812	0.6019	0.7964	0.5938	0.8211	0.612
